# Evaluation of Structural Retinal Layer Alterations in Retinitis Pigmentosa

**DOI:** 10.22336/rjo.2023.54

**Published:** 2023

**Authors:** Kamil Yavuzer, Mehmet Citirik, Beyza Yavuzer

**Affiliations:** * Dunya Goz Hospital, Gaziantep, Turkey; ** University of Health Sciences, Ankara Etlik City Hospital, Ankara, Turkey; *** University of Health Sciences, Van Training and Research Hospital, Van, Turkey

**Keywords:** choroid, optical coherence tomography, optical coherence tomography angiography, retinal segmentation analysis, retinitis pigmentosa

## Abstract

**Objective:** This study aimed to analyze retinal layers in the macular region using spectral-domain optical coherence tomography (SD-OCT). Additionally, we examined the retinal vascular plexus densities of the eyes using optical coherence tomography angiography (OCT-A), specifically in patients with retinitis pigmentosa (RP).

**Methods:** In the study, 36 eyes from patients with retinitis pigmentosa (RP) and 36 eyes from healthy controls were included. Measurements involved assessing the thicknesses of each retinal layer at the central fovea, parafoveal, and perifovea using spectral domain optical coherence tomography (SD-OCT). Moreover, the study involved the evaluation of retinal capillary plexus densities (RCPD), encompassing deep capillary plexus density values, superficial capillary plexus, and radial peripapillary capillary plexus. This assessment was performed using optical coherence tomography angiography (OCT-A).

**Results:** No statistically significant difference in retinal thickness was found in the central fovea between the two groups. The thicknesses of the INL, OPL, and PRL in the parafoveal regions as well as the RPE in the perifoveal regions increased in the RP group. Nonetheless, the ONL, IPL, GCL, and RNFL demonstrated reduced thickness in both the perifoveal and parafoveal areas. The OCT-A findings indicated that patients with RP exhibited lower values for all RCPD.

**Conclusion:** The retinal layers and RCPD were significantly impacted at varying rates of RP. It is essential to acknowledge that this alteration may be significant in the context of the retinal findings in patients with RP.

**Abbreviations:** SD-OCT = Spectral-domain optical coherence tomography, OCT-A = Optical coherence tomography angiography, RP = Retinitis pigmentosa, ETDRS = Early Treatment Diabetic Retinopathy Study, SD = standard deviation, TRT = Total Retinal Thickness, IRT = Inner Retinal Thickness, ORT = Outer Retinal Thickness, RNFL = Retinal Nerve Fiber Layer, GCL = Ganglion Cell Layer, IPL = Inner Plexiform Layer, INL = inner nuclear layer, OPL = Outer Plexiform Layer, ONL = Outer Nuclear Layer, PRL = Photoreceptor layer, RPE = Retinal Pigment Epithelium, µm = micrometer, PaFoSu = parafovea superior, PeFoSu = perifovea superior, PaFoNa = parafovea nasal, PeFoNa = perifovea nasal, PaFoTe = parafovea temporal, PeFoTe = perifovea temporal, PaFoIn = parafovea inferior, PeFoIn = perifovea inferior

## Introduction

Retinitis pigmentosa (RP), the most prevalent type of hereditary retinal dystrophy, is characterized by gradual deterioration and eventual demise of the retinal photoreceptors [**1**]. With an estimated prevalence of around 1 in 4,000 individuals, retinitis pigmentosa (RP) is the most common type of rod-cone dystrophy. The initial indication is night blindness, followed by a gradual reduction in peripheral vision during daylight, ultimately culminating in blindness [**2**]. Retinitis pigmentosa (RP) is inherently highly heterogeneous. Mutations in more than 50 genes are known to cause nonsyndromic RP. Approximately 3,100 mutations have been reported [**3**-**6**].

Previous studies on RP have reported that the earliest histopathological alterations involved the shortening of the photoreceptor outer segments [**7**-**9**]. The onset of this change is initiated in the mid-periphery and advances toward the central retina. Therefore, morphological evaluation of retinal changes may be useful. Functional assessment can also play a crucial role in the prediction of disease progression. Additionally, assessing residual retinal function in patients is important for a comprehensive understanding of their condition [**10**]. Optical coherence tomography (OCT) quantitatively monitors morphological changes in the retinal tissue. The updated OCT software facilitates more straightforward and precise automatic differentiation, as well as thickness measurements for each retinal layer [**11**]. Optical coherence tomography angiography (OCT-A) has served as a valuable guide for researchers, particularly in patients with RP, by offering information on the vascular densities of retinal vessels [**12**]. Examining the retinal layers individually and acquiring information about vascular densities in RP can enhance our understanding of disease progression and clinical manifestations. In this study, the analysis focused on individual examination of macular retinal layers in eyes affected by retinitis pigmentosa (RP). The objective of this study was to assess the retinal vascular plexus density and evaluate the impact of pathophysiological processes on the retina.

## Patients and methods

This was a retrospective case-control study. Approval was obtained from the local ethics committee and the principles of the Declaration of Helsinki were followed. 

The 36 eyes (20 patients) showing the classical triad of RP were included in this study, all of which underwent retinal segment analysis and OCT-A. The study also incorporated a control group comprising 36 eyes of 18 patients.

The study excluded patients with systemic diseases such as hypertension or diabetes mellitus; ocular conditions requiring chronic medication use such as glaucoma, uveitis, or dry eye; individuals with a history of intraocular trauma or surgery; and patients with additional findings affecting the macular area. Additional findings included macular atrophy, epiretinal membrane, vitreomacular adhesion/traction, or cystoid macular edema. The study excluded the eyes of patients with RP with additional pathologies affecting the macular region. Instances with measurement artifacts and cases in which retinal segmentation analysis could not be performed were excluded from the study.

Each patient underwent a full ocular examination. Fundus examination was conducted following pupil dilatation, initiated after the slit-lamp examination. Fundus photographs of seven regions were taken using a digital retinal camera system (Zeiss Visucam NM Pro Fundus Camera; ZEISS, Jena, Germany). 

OCT-A was performed using AngioVue OCT-A (RTVue XR Avanti version 2017.1.0.151; Optovue, Inc., Fremont, CA, USA). Density values for the deep capillary plexus (DCP), superficial capillary plexus (SCP), and radial peripapillary capillary plexus (RPCP) were calculated. All OCT-A images were consistently measured by the same individual (KY), with careful attention paid to ensure that the signal strength index was equal to or greater than seven. Low-quality images, characterized by a signal strength of less than seven, the presence of one or more blinks, poor fixation, and segmentation errors leading to motion or double artifacts, were excluded from the study.

**Fig. 1:**
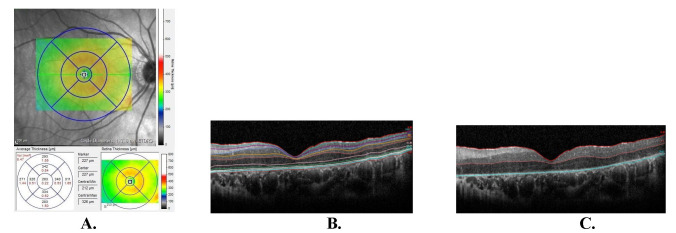
**A** Nine regions of the macular retina map defined by the Early Treatment Diabetic Retinopathy Study (ETDRS) grid; **B** OCT image showing all retinal layers in a patient with retinitis pigmentosa; **C** Inner and outer retinal layers. The part from the inner retina from the ILM to the border between INL-OPL. Outer retina from the INL-OPL border to the RPE

SD-OCT was used to measure the total retinal thickness and the thickness of the retinal layers in the macular region (HRA2-Heidelberg Retina Angiography, Optical Coherence Tomography, Heidelberg Engineering, Heidelberg, Germany). SD-OCT was conducted on sections measuring 6 × 6 mm² and centered on the fovea. Two types of data were collected: 1) numerical data displaying the average retinal thickness in the specified area of interest, and 2) color-coded images facilitating comparison with normative data. An Early Treatment Diabetic Retinopathy Study (ETDRS) grid was employed to identify nine regions on the SD-OCT retinal map. According to the definition, the foveal area was delineated by a central circle with a diameter of 1 mm centered on the foveola. The parafoveal region was designated as a two-millimeter diameter surrounding the circle, while the perifoveal region was defined as the area within the three-millimeter diameter surrounding the last circle (**Fig. 1 A**). Automatic segmentation of macular retinal layers (**Fig. 1 B**) encompassed the retinal nerve fiber layer (RNFL), ganglion cell layer (GCL), inner plexiform layer (IPL), inner nuclear layer (INL), outer plexiform layer (OPL), outer nuclear layer (ONL), photoreceptor layer (PRL), and retinal pigment epithelium (RPE). The combined thickness of the RNFL + GCL + IPL + INL was referred to as the Inner Retinal Thickness (IRT), whereas the combined thickness of the OPL + ONL + PRL was termed the Outer Retinal Thickness (ORT) (**Fig. 1 C**).

**Statistical analysis**

Patient data were entered into SPSS 18.0 (SPSS Inc., Chicago, Illinois, USA). The Shapiro-Wilk test was used to evaluate the normal distribution of the data. For normally distributed data, an independent group t-test was used to compare parameters obtained from the groups. Statistical significance was determined when the p-value was < 0.05.

## Results

The RP group consisted of 36 eyes of 20 patients, whereas the control group consisted of 36 eyes of 18 patients. The mean age of patients in the RP group was 33.47 ± 15.96 years, and in the control group, it was 37.00 ± 13.84 years. In Group 1, the male-to-female ratio was 12:8, whereas in group 2, it was 10:8. No statistically significant differences were observed in the demographic characteristics between the two groups (p > 0.05). 

Retinal segmentation analysis was utilized to assess the retinal layers, and the findings are presented in **Tables 1** and **2**.

**Table 1:** Data in both groups of RNFL, GCL, IPL, INL, OPL, ONL, PRL, and RPE layers

Layers	Retinal Region	Retinitis Pigmentosa, Average values ± SD (µm)	Control Group, Mean values ± SD (µm)	p-value
RNFL	Central	11.69 ± 3.25	12.83 ± 3.05	0.214
	PaFoSu	19.34 ± 9.33	24.67 ± 4.36	<0.001
	PeFoSu	28.39 ± 12.37	39.03 ± 7.97	0.035
	PaFoNa	18.77 ± 5.91	22.44 ± 5.82	0.091
	PeFoNa	31.97 ± 13.68	48.94 ± 7.81	0.012
	PaFoTe	16.23 ± 4.99	17.89 ± 2.94	<0.001
	PeFoTe	16.57 ± 5.04	19.42 ± 2.27	<0.001
	PaFoIn	20.83 ± 10.21	25.61 ± 4.60	<0.001
	PeFoIn	26.73 ± 10.62	39.81 ± 6.49	0.053
GCL	Central	16.54 ± 7.25	16.86 ± 7.28	0.518
	PaFoSu	29.54 ± 19.99	51.31 ± 6.80	<0.001
	PeFoSu	24.39 ± 9.36	36.19 ± 4.80	<0.001
	PaFoNa	33.63 ± 18.62	50.22 ± 6.41	<0.001
	PeFoNa	27.00 ± 9.14.	38.39 ± 4.52	<0.001
	PaFoTe	33.69 ± 18.27	46.25 ± 6.12	<0.001
	PeFoTe	22.29 ± 13.18	37.50 ± 5.21	<0.001
	PaFoIn	28.14 ± 18.63	50.56 ± 7.70	<0.001
	PeFoIn	22.45 ± 7.19	35.56 ± 4.70	0.003
IPL	Central	20.31 ± 5.69	21.92 ± 5.22	0.444
	PaFoSu	28.06 ± 10.89	40.08 ± 4.72	<0.001
	PeFoSu	28.94 ± 4.70	28.94 ± 3.08	0.123
	PaFoNa	30.69 ± 11.06	41.42 ± 4.84	<0.001
	PeFoNa	32.43 ± 5.44	29.61 ± 3.37	0.098
	PaFoTe	30.20 ± 11.90	40.36 ± 4.84	<0.001
	PeFoTe	22.43 ± 7.84	33.25 ± 3.04	<0.001
	PaFoIn	27.92 ± 11.13	40.14 ± 4.69	<0.001
	PeFoIn	27.48 ± 3.80	28.44 ± 3.44	0.964
INL	Central	23.86 ± 6.12	20.17 ± 6.68	0.715
	PaFoSu	51.40 ± 9.67	42.42 ± 3.34	<0.001
	PeFoSu	34.00 ± 6.54	33.11 ± 2.93	<0.001
	PaFoNa	49.11 ± 9.24	41.00 ± 4.28	<0.001
	PeFoNa	37.11 ± 6.84	34.69 ± 2.78	0.001
	PaFoTe	45.09 ± 9.61	37.53 ± 3.56	<0.001
	PeFoTe	33.09 ± 4.32	34.22 ± 2.82	0.030
	PaFoIn	52.08 ± 10.66	41.00 ± 3.76	<0.001
	PeFoIn	33.36 ± 5.90	32.69 ± 3.29	0.006
OPL	Central	29.40 ± 9.78	26.86 ± 12.10	0.982
	PaFoSu	40.74 ± 8.32	36.42 ± 9.09	0.650
	PeFoSu	32.52 ± 4.53	28.86 ± 3.31	0.139
	PaFoNa	41.94 ± 10.16	33.47 ± 7.30	0.211
	PeFoNa	34.86 ± 5.36	29.64 ± 3.07	0.003
	PaFoTe	35.91 ± 7.47	31.42 ± 5.77	0.042
	PeFoTe	32.00 ± 5.35	27.75 ± 2.16	<0.001
	PaFoIn	38.19 ± 9.03	32.72 ± 6.42	0.028
	PeFoIn	32.52 ± 5.04	28.28 ± 4.21	0.146
ONL	Central	75.89 ± 19.14	88.94 ± 15.45	0.081
	PaFoSu	54.17 ± 17.57	66.89 ± 16.64	0.739
	PeFoSu	42.88 ± 16.56	59.31 ± 9.70	0.001
	PaFoNa	57.06 ± 17.36	72.97 ± 12.95	0.067
	PeFoNa	41.36 ± 15.13	56.17 ± 8.31	0.009
	PaFoTe	55.03 ± 18.39	73.67 ± 10.52	0.008
	PeFoTe	43.69 ± 16.79	58.81 ± 8.06	<0.001
	PaFoIn	55.50 ± 14.49	69.22 ± 10.07	0.119
	PeFoIn	39.97 ± 15.10	53.75 ± 7.51	<0.001
PRL	Central	80.92 ± 3.97	87.19 ± 3.85	0.583
	PaFoSu	83.94 ± 12.38	80.94 ± 3.03	0.006
	PeFoSu	81.16 ± 8.36	79.19 ± 2.79	<0.001
	PaFoNa	80.50 ± 3.68	82.06 ± 3.05	0.584
	PeFoNa	80.22 ± 5.31	78.56 ± 2.49	0.037
	PaFoTe	79.72 ± 4.69	80.94 ± 2.62	0.046
	PeFoTe	81.28 ± 11.97	77.92 ± 2.87	<0.001
	PaFoIn	80.89 ± 3.89	79.75 ± 3.37	0.252
	PeFoIn	79.80 ± 6.39	76.67 ± 2.48	0.001
RPE	Central	14.17 ± 2.40	16.69 ± 1.91	0.223
	PaFoSu	16.03 ± 12.67	14.89 ± 1.92	0.035
	PeFoSu	15.75 ± 6.55	13.47 ± 1.28	<0.001
	PaFoNa	13.06 ± 1.66	15.11 ± 1.85	0.325
	PeFoNa	13.81 ± 3.73	13.28 ± 1.32	0.013
	PaFoTe	13.11 ± 2.53	13.94 ± 1.35	0.079
	PeFoTe	16.28 ± 10.51	12.50 ± 1.54	<0.001
	PaFoIn	12.97 ± 1.50	14.25 ± 1.83	0.064
	PeFoIn	14.31 ± 5.04	12.92 ± 1.18	<0.001
SD = Standard Deviation, RNFL = Retinal Nerve Fiber Layer, GCL = Ganglion Cell Layer, IPL = Inner Plexiform Layer, INL = inner nuclear layer, OPL = Outer Plexiform Layer, ONL = Outer Nuclear Layer, PRL = Photoreceptor layer, RPE = Retinal Pigment Epithelium, µm = micrometer, PaFoSu = parafovea superior, PeFoSu = perifovea superior, PaFoNa = parafovea nasal, PeFoNa = perifovea nasal, PaFoTe = parafovea temporal, PeFoTe = perifovea temporal, PaFoIn = parafovea inferior, PeFoIn = perifovea inferior.				

**Table 2:** Data in both groups of TRK, IRT, and ORT ratios

Layers	Retinal Region	Retinitis Pigmentosa, Average values ± SD (µm)	Control Group, Mean values ± SD (µm)	p-value
TRT	Central	255.50 ± 35.92	270.89 ± 28.01	0.238
	PaFoSu	304.72 ± 38.96	340.28 ± 24.94	0.004
	PeFoSu	271.16 ± 32.871	304.22 ± 17.64	<0.001
	PaFoNa	309.28 ± 40.34	343.50 ± 18.31	0.001
	PeFoNa	282.17 ± 35.72	315.89 ± 15.02	<0.001
	PaFoTe	293.00 ± 38.09	327.92 ± 16.74	<0.001
	PeFoTe	250.28 ± 35.23	288.75 ± 12.50	<0.001
	PaFoIn	302.39 ± 35.79	339.19 ± 18.03	0.002
	PeFoIn	260.06 ± 32.96	295.00 ± 17.19	<0.001
IRT	Central	72.40 ± 17.28	71.78 ± 20.77	0.443
	PaFoSu	128.34 ± 34.49	158.47 ± 14.02	<0.001
	PeFoSu	115.71 ± 25.55	137.28 ± 13.73	<0.001
	PaFoNa	132.20 ± 32.46	155.08 ± 13.97	<0.001
	PeFoNa	128.51 ± 21.86	151.64 ± 11.35	<0.001
	PaFoTe	125.20 ± 30.51	142.03 ± 13.88	<0.001
	PeFoTe	94.37 ± 26.42	124.39 ± 10.39	<0.001
	PaFoIn	128.97 ± 31.59	157.31 ± 17.54	<0.001
	PeFoIn	110.03 ± 19.72	136.50 ± 10.33	0.001
ORT	Central	186.39 ± 26.41	203.00 ± 13.13	<0.001
	PaFoSu	177.72 ± 22.32	184.25 ± 12.74	0.012
	PeFoSu	155.53 ± 20.43	167.36 ± 9.85	<0.001
	PaFoNa	178.33 ± 21.80	188.50 ± 14.17	0.051
	PeFoNa	155.47 ± 19.27	164.36 ± 8.91	0.002
	PaFoTe	169.67 ± 23.38	186.03 ± 12.14	0.006
	PeFoTe	156.08 ± 26.57	164.47 ± 10.02	<0.001
	PaFoIn	174.58 ± 18.29	181.69 ± 14.24	0.338
	PeFoIn	150.43 ± 21.91	158.69 ± 10.91	0.002
SD = standard deviation, TRT = Total Retinal Thickness, IRT = Inner Retinal Thickness, ORT = Outer Retinal Thickness, SD = standard deviation, µm = micrometer, PaFoSu = parafovea superior, PeFoSu = perifovea superior, PaFoNa = parafovea nasal, PeFoNa = perifovea nasal, PaFoTe = parafovea temporal, PeFoTe = perifovea temporal, PaFoIn = parafovea inferior, PeFoIn = perifovea inferior				

In the central 1 mm foveal area, the RP group exhibited thicker INL, OPL, and IRT than the control group, but this difference was not statistically significant (p > 0.05). The ORT was significantly lower (P < 0.001). The RP group had thinner RNFL, GCL, IPL, ONL, PRL, RPE, and TRT than the control group; however, this difference was not statistically significant (p > 0.05).

Analyses were performed in four regions (nasal, temporal, inferior, and superior) of the parafoveal region. In the parafovea superior region, the RP, INL, PRL, and RPE were significantly thicker (p < 0.05). RNFL, GCL, IPL, TRT, IRT, and ORT were significantly thinner (p < 0.05). The INL and OPL were significantly higher in the RP group (p < 0.05). RNFL, GCL, IPL, TRT, and IRT were significantly thinner (p < 0.05). In the parafoveal nasal region, the INL was significantly thicker in the RP group (P < 0.001). GCL, IPL, TRT, and IRT scores were significantly lower (p < 0.05). In the parafovea temporal region, the RP, INL, and OPL were significantly thicker (p < 0.001 and P = 0.042, respectively). The RNFL, GCL, IPL, ONL, PRL, TRT, IRT, and ORT were all found to be statistically significantly thinner (p < 0.05).

Analyses were performed in four regions (nasal, temporal, inferior, and superior) of the perifoveal region. In the perifovea superior region, the RP, INL, PRL, and RPE were significantly thicker (p < 0.05). RNFL, GCL, ONL, TRT, IRT, and ORT were significantly lower (p < 0.05). The INL, PRL, and RPE were significantly thicker in the inferior perifovea region of the RP group (p < 0.05). GCL, ONL, TRT, IRT, and ORT were significantly lower (P < 0.05). The INL, OPL, PRL, and RPE were significantly higher in the RP of the perifoveal nasal region (p < 0.05). RNFL, GCL, ONL, TRT, IRT, and ORT were significantly lower (p < 0.05). In the perifovea temporal region, the OPL, PRL, and RPE showed statistically significant increases in thickness in the RP group (P < 0.05). Conversely, RNFL, GCL, IPL, INL, ONL, TRT, IRT, and ORT were significantly lower in the RP group (p < 0.05). 

In the RP group, the OCT-A-detected density of RPCP was 45.57 ± 1.82%, whereas in the control group, it was 49.46 ± 2.78%. The RP group exhibited an SCP density of 42.62% ± 3.18% and a DCP density of 41.33% ± 1.33%. Conversely, the control group demonstrated an elevated SCP density of 48.02% ± 1.34% and a greater DCP density of 49.34% ± 2.51%. In the RP group, the RPCP, SCP, and DCP densities were comparatively lower (P = 0.044, P < 0.001, and P = 0.016, respectively).

## Discussion

The attenuation of retinal vessels occurs before the formation of bone spicule pigments in the RP [**13**]. Histopathological investigations revealed that the migration of retinal pigment epithelium (RPE) cells around the inner retinal blood vessels triggers the accumulation of extracellular matrix (ECM). This accumulation resembled the formation of an ectopic Bruch’s membrane. Over time, this perivascular ECM gradually thickens, leading to occlusion of the vessel lumen [**14**]. The inner and outer retinal segments are supplied by distinct vascular structures; hence, circulatory disorders may affect the different retinal segments. Vascular pathologies are prevalent in many retinal diseases because of the abundance of vascular tissue in the retina, vascular pathologies being prevalent in many retinal diseases [**15**]. In this study, we investigated the effect of RP on retinal perfusion and retinal layers in patients, a condition that may involve a complex genetic structure.

There were four plexuses for retinal vascularization. The RPCP supplies RNFL. The SCP feeds into the GCL and IPL. The ICP supplies the IPL and INL. The DCP feeds the INL and OPL [**16**]. The outer retinal layers are supplied with nutrients through diffusion from the choroid [**17**]. OCT-A studies in eyes with RP revealed a lower density of RPCP, SCP, and DCP than in controls [**18**]. In a study conducted by Jauregui et al. [**13**], which examined the progression of patients with RP over one year, decreases in SCP and DCP densities were reported. Interestingly, no observable changes in choriocapillaris blood flow were observed. Koyanagi et al. [**19**] reported that in patients with RP, the SCP was decreased (p = 0.049), but no significant change was observed in the DCP) (p = 0.757) in the central 1 mm foveal area. In the same study, it was reported that vascular densities in both the SCP and DCP decreased in the parafoveal area of patients with RP. However, the DCP was found to be less affected than the SCP [**19**]. These studies demonstrated a reduction in RPCP, SCP, and DCP densities in patients with RP, with DCP being relatively less affected. The OCT-A findings in our study were consistent with those previously reported in the literature. We also noted a decrease in the densities of DCP, SCP, and RPCP in the RP group. In our study, the absence of a significant difference in layer thickness between the groups, particularly in the central 1 mm area, suggested that the foveal central vascular structure might have been relatively protected from the pathological process compared to the parafoveal region.

In our study, patients with RP had thinner inner retinal layers including the RNFL, GCL, and IPL. We hypothesized that the thinning might have been attributed to a reduction in vascular flow in the RPCP and SCP. Many studies have focused primarily on the peripapillary RNFL in the context of RP. While the majority of these studies emphasize an increase in the peripapillary RNFL, some studies have reported a decrease in the peripapillary RNFL. The variability in findings highlights the complexity of retinal changes associated with RP and underscores the need for comprehensive investigation and analysis [**20**-**22**]. Yoon et al. [**23**] reported that the macular RNFL was thicker in RP patients compared to the control group. In the same study, it was noted that the ganglion cell-inner plexiform layer (GCL-IPL) complex was thicker in the RP group than in the control group. In their study, the thickness measurements were compared along a horizontal or vertical line. In our study, the comparison was conducted using a 6 × 6 mm² macular map; thus, we considered the results to be more reliable. A study that assessed the thickness of the GCL using a methodology similar to ours reported that the GCL was thinner in RP patients. Furthermore, this thinning was correlated with a decrease in SCP density [**24**].

Despite progressive thinning of the ONL in RP, studies have indicated that the inner retinal layers remain histologically intact [**25**]. Morphological studies have indicated a lower degree of cellular loss in the INL than in the photoreceptor layer [**26**]. Sayo et al. [**27**] reported that the INL was thicker in patients with RP than in controls. In our study, we observed that the INL was thicker in the eyes with RP. We hypothesized that this observation might have been attributed to the genetic pathophysiological mechanism of the disease and the relatively less affected density of the DCP. Additionally, in our study, we found that the OPL was thicker in eyes with RP. The OPL receives nourishment from dual feeding of DCP and the choriocapillaris. This dual feeding suggests that the OPL may have a protective mechanism against the pathological processes of RP. To the best of our knowledge, no study has directly analyzed the thickness of the OPL in eyes with RP.

In RP, the progression of degeneration typically begins with the disappearance of the outer segments of photoreceptors. Subsequently, there is a loss of the inner segments, and ultimately, the photoreceptor nuclei are lost. This sequence of events reflects the characteristic pattern of photoreceptor degeneration in RP [**28**]. Given that the outer nuclear layer comprises cone and rod nuclei, the ONL thickness tends to decrease in RP [**29**].

Although this reduction has often been linked to X-linked RP, it is likely present in most forms of RP. In our study, ONL thinning was observed in the ONL of all patients with RP; however, genetic research was not conducted as part of this study. Retinal remodeling initiates following the loss of photoreceptor outer segments. The RPE cells are also involved in this process. In the context of eye pathology, bone spicules are deposits of pigment granules that occupy the retina. These structures often originate from RPE cells surrounding blood vessels. The appearance of bone spicules is a characteristic feature of certain retinal disorders including retinitis pigmentosa. Photoreceptor damage, gliosis (proliferation of glial cells), ONL ablation, RPE migration, and the subsequent remodeling phase collectively contribute to irregularities in the outer segments of the retina [**30**]. In our study, certain regions of the macular map were thicker in the RP group, whereas other regions were thicker in the control group. In contrast to other layers, the uneven distribution of these layer thicknesses can be explained by the irregularity of the retinal remodeling process.

A notable strength of this study was the indirect assessment of IRT perfusion by evaluating the SCP and DCP using OCT-A. This study had certain limitations. First, genetic analysis was not conducted, which could have provided valuable insights into the underlying genetic factors associated with retinal changes observed in patients with retinitis pigmentosa (RP). Choroidal imaging was not performed in the enhanced depth imaging (EDI) mode. Previous studies have indicated choroidal thinning in eyes with RP, and incorporating this aspect of analysis could have provided a more comprehensive understanding of ocular changes in study participants [**31**].

## Conclusion

The results of this study indicated that there was no significant difference in retinal layer thickness among patients with RP of the central fovea. However, notable thinning was observed in the ONL, IPL, GCL, and RNFL in both parafoveal and perifoveal regions. OCT-A revealed that patients with RP had a lower vascular density in the DCP, SCP, and RCP layers. In addition to the genetic pathophysiological processes of RP, this process also affects retinal vascular perfusion.

**Conflict of Interest Statement**

The authors declare that they have no conflict of interest.

**Informed Consent and Human and Animal Rights Statement**

Informed consent was obtained from all the individuals included in this study.

**Authorization for the use of human subjects**

Ethical approval: The research related to human use complied with all relevant national regulations and institutional policies, followed the tenets of the Helsinki Declaration, and was approved by the Ethics Committee of Van Training and Research Hospital, Van, Turkey (No:2020/15).

**Acknowledgments**

None.

**Sources of Funding**

This study did not receive any specific grants from funding agencies in the public, commercial, or not-for-profit sectors.

**Disclosures**

None.

**Contribution**

All the authors of the manuscript have equal contributions.

**Presentation to an event**

The content and summary of this study were presented at the EuRetina 2021 conference (https://euretina.org/resource/abstract_2021_evaluation-of-retinal-layer-alterations-in-retinitis-pigmentosa-with-retinal-segmentation-analysis/).
